# Sample pooling: burden or solution?

**DOI:** 10.1016/j.cmi.2021.04.007

**Published:** 2021-04-18

**Authors:** Nadja Grobe, Alhaji Cherif, Xiaoling Wang, Zijun Dong, Peter Kotanko

**Affiliations:** 1) Renal Research Institute, New York, NY, USA; 2) New York University College of Arts and Science, New York, NY, USA; 3) Icahn School of Medicine at Mount Sinai, New York, NY, USA

**Keywords:** Antibody, Antigen, COVID-19, Pool testing, RT-PCR, SARS-CoV-2, Viral

## Abstract

**Background:**

Pool-testing strategies combine samples from multiple people and test them as a group. A pool-testing approach may shorten the screening time and increase the test rate during times of limited test availability and inadequate reporting speed. Pool testing has been effectively used for a wide variety of infectious disease screening settings. Historically, it originated from serological testing in syphilis. During the current coronavirus disease 2019 (COVID-19) pandemic, pool testing is considered across the globe to inform opening strategies and to monitor infection rates after the implementation of interventions.

**Aims:**

This narrative review aims to provide a comprehensive overview of the global efforts to implement pool testing, specifically for COVID-19 screening.

**Sources:**

Data were retrieved from a detailed search for peer-reviewed articles and preprint reports using Medline/PubMed, medRxiv, Web of Science, and Google up to 21st March 2021, using search terms “pool testing”, “viral”, “serum”, “SARS-CoV-2” and “COVID-19”.

**Content:**

This review summarizes the history and theory of pool testing. We identified numerous peer-reviewed articles that describe specific details and practical implementation of pool testing. Successful examples as well as limitations of pool testing, in general and specifically related to the detection of severe acute respiratory syndrome coronavirus 2 (SARS-CoV-2) RNA and antibodies, are reviewed. While promising, significant operational, pre-analytical, logistical, and economic challenges need to be overcome to advance pool testing.

**Implications:**

The theory of pool testing is well understood and numerous successful examples from the past are available. Operationalization of pool testing requires sophisticated processes that can be adapted to the local medical circumstances. Special attention needs to be paid to sample collection, sample pooling, and strategies to avoid re-sampling.

## Introduction

### History

In 1943 the economist Robert Dorfman first developed the theory and practice of pool testing to detect syphilis (based on the serological Wasserman test) in US soldiers during World War II [1]. In his seminal paper, Dorfman addressed key questions regarding resource savings and efficient group testing size. A key insight of his analysis was that the optimal pool (group) size depends on the disease prevalence. The consequences of test sensitivity and sample dilution were addressed later by Hwang [2] and by Wein and Zenios [3]. Pooling strategies have also been widely used for the detection of pathogens including hepatitis B and C viruses [4,5], HIV [6], and *Neisseria gonorrhoeae* [7]. More recently, pool testing has been advocated to address testing resource constraints during the ongoing coronavirus disease 2019 (COVID-19) pandemic [8,9].

### Use cases related to COVID-19

Pool testing for COVID-19 has recently gained attention since it can increase testing capacity, lower the cost per test, and conserve reagents during times of sudden and heavy inflow of test requests, pandemic surveillance, large-scale population testing, clearing of backlogs, and in settings with limited resources. A pool-testing strategy is especially useful to screen for asymptomatic COVID-19 patients/cases and to assist with prevalence estimation, thereby informing policy-makers, reducing transmission, and alleviating the strain on healthcare services. It could become a particularly important tool to longitudinally monitor the effectiveness of contact reduction measures and to inform reopening strategies after lockdowns. Pool-testing strategies are used to screen groups at risk, such as the elderly, patients with underlying medical conditions, or professional groups with a low pre-test probability who are exposed to the virus while also posing a risk of spreading it (such as healthcare workers, emergency responders, essential workers, factory shift workers, or individuals working in transportation or delivery services). Pool testing for COVID-19 has received emergency use authorization from the U.S. Food and Drug Administration in July 2020. Countries in Asia, Europe, North America, South America, Africa, and Oceania have already implemented this strategy in communities such as universities, hospitals, care homes, etc. (Table 1).

### Mathematics of pool-testing strategies

**Fig. 1.**
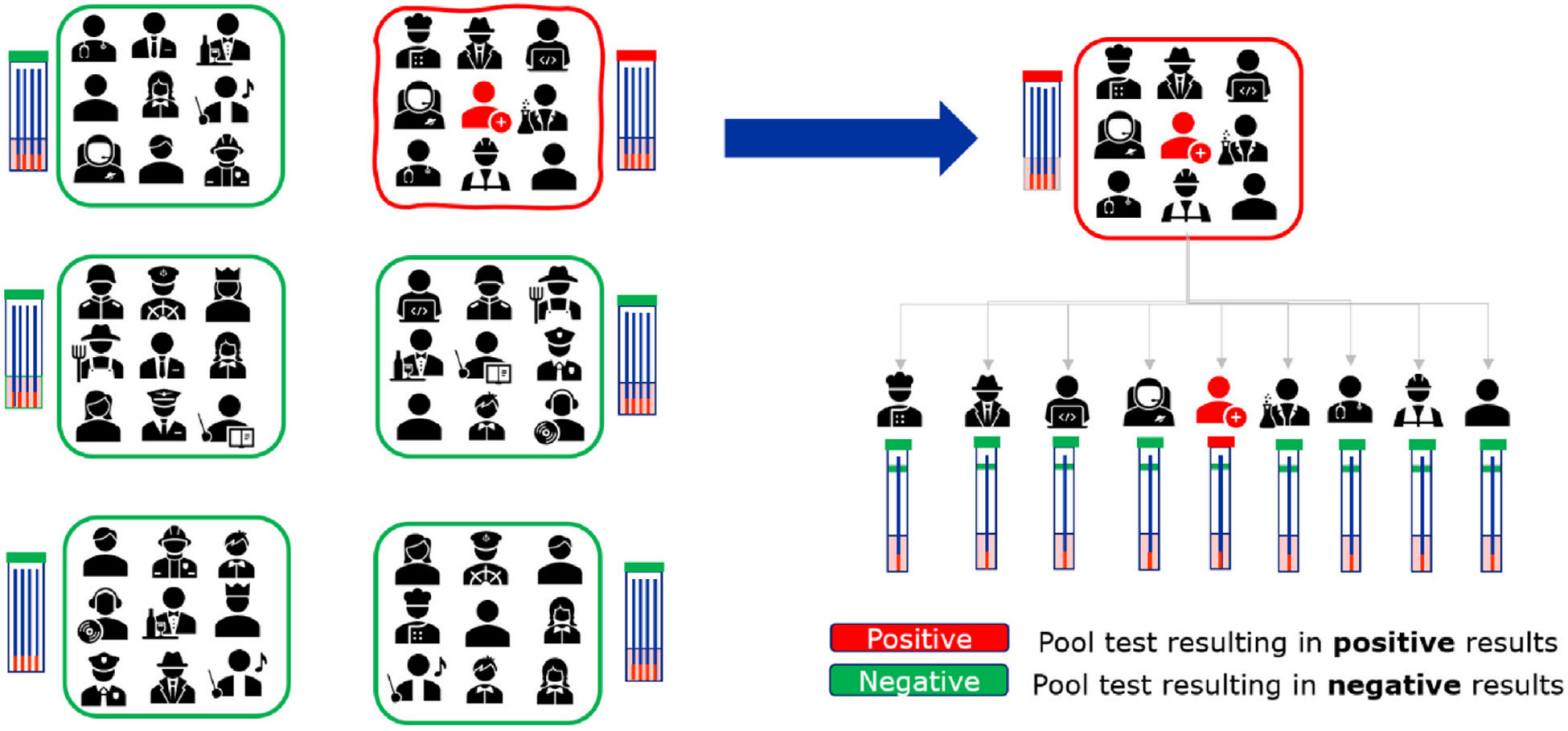
Pool testing principle. Samples are pooled and each pool is tested. If a pool tests negative, as indicated in green, the test is complete. If the pool tests positive, as indicated in red, all samples of that pool are tested individually to identify the sample(s) that contributed to the positive pool.

**Fig. 2.**
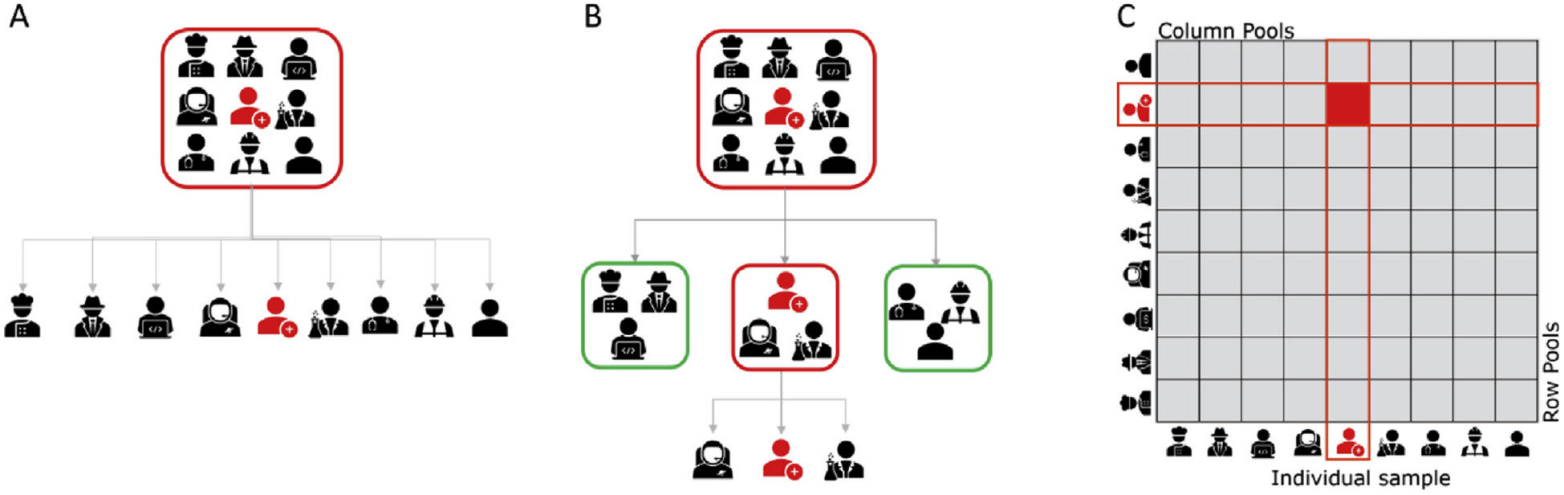
Pool-testing strategies. (A) Two-stage hierarchical pool testing consists of initial testing of a pool followed by individual testing if the pool tests positive. (B) Three-stage hierarchical pool testing first tests a pool and, if positive, is followed by testing of non-overlapping sub-pools and individual testing of positive sub-pools. (C) Matrix-based non-hierarchical pool testing uses a combinatorial pool-testing approach.

Pool or group testing is the amalgamation of samples into a group or pool which is tested as if it were a single sample. The basic assumption of group testing is that it is possible to perform simultaneous tests for any finite subset with two categories (i.e. healthy or defective, success or failure). Dorfman proposed the first known use of pool testing as a cost-efficient alternative to testing individual samples [1]. If a group tests negative, then each member is declared negative. However, if the group tests positive, each member of the group would be retested individually because at least one member of the group is positive, but how many and which one(s) are unknown (Fig. 1). Several pooling alternatives have been proposed (Fig. 2), and they often fall into one of two classes: namely hierarchical (Fig. 2A,B) and non-hierarchical (Fig. 2C) group testing [10-12]. Hierarchical group or adaptive testing strategies refer to a strategy in which group testing is performed in stages. In these strategies, individuals are (re)tested depending on the results of the preceding stage and only once per stage. For example, two-stage hierarchical pool testing (S2) consists of initial testing of a pool followed by individual testing if the pool tests positive (Fig. 2A). Three-stage hierarchical pool testing (S3) first tests a pool and, if positive, this is followed by testing of non-overlapping sub-pools and individual testing of positive sub-pools (Fig. 2B). Hence, Dorfman's pool testing can be seen as a two-stage strategy, and more stages can be included, as in binary splitting or multistage pool testing.

Alternative to hierarchical pool testing, non-hierarchical, non-adaptive, or combinatorial group testing refers to strategies in which the same individuals are tested multiple times within each stage. This is usually facilitated by the use of an array-like or matrix-like arrangement (M2) of the samples, in which pools are made from all samples in the same row or the same columns. The k-column and k-row pools are tested simultaneously so that each sample is tested twice in the first stage (Fig. 2C). Therefore, the pool size of the M2 test is defined as the square of the sample number in each pool. As in the hierarchical pool strategies, if individuals are declared negative, then the corresponding row and column groups are negative. However, if there is a positive result, then all positive samples will lie at the intersection of a positive row and positive column pool. These samples are then re-tested individually to identify positive individuals, where some or all may be positive [11].

As in hierarchical pool testing, non-hierarchical pool strategies can also be augmented into multi-stages or higher dimensional strategies. For example, instead of first using array-like arrangement to test samples, one could first test a master pool. The master pool size can be determined similarly to the optimal pool size of the S2 test, or k^2^ specimens can be placed on a *k* × *k* matrix. If the master pool is positive, then combinatorial pool testing M2 can be performed. This approach is known as an array-like arrangement with master pool (M2m). A recent review by Lapogati and colleagues describes the different pooling strategies in more detail [13]. Research in group testing has recently gained attention in classification and estimation literature. Here, we focus on the classification aspect of group testing, which is more relevant to addressing testing shortage issues.

The classification problem is concerned with the identification of all positives among those individuals tested by re-testing individuals belonging to pools which test positive. For a classification problem, both hierarchical and non-hierarchical matrix-based pool-testing strategies are much preferable to individual testing, especially under limited testing capability.

Cherif et al. [14] showed that, for S2 with different sensitivity values, the efficiency of pool testing is diminished as the prevalence approaches 30%, beyond which pool testing is no longer advantageous compared with individual testing. Among the group-testing strategies, the best pool-testing method may differ depending on the various characteristics, ranging from the prevalence, sensitivity and specificity, to resource constraint. The gains in efficiency due to pool testing are substantially better at lower prevalence [15-18].

**Fig. 3.**
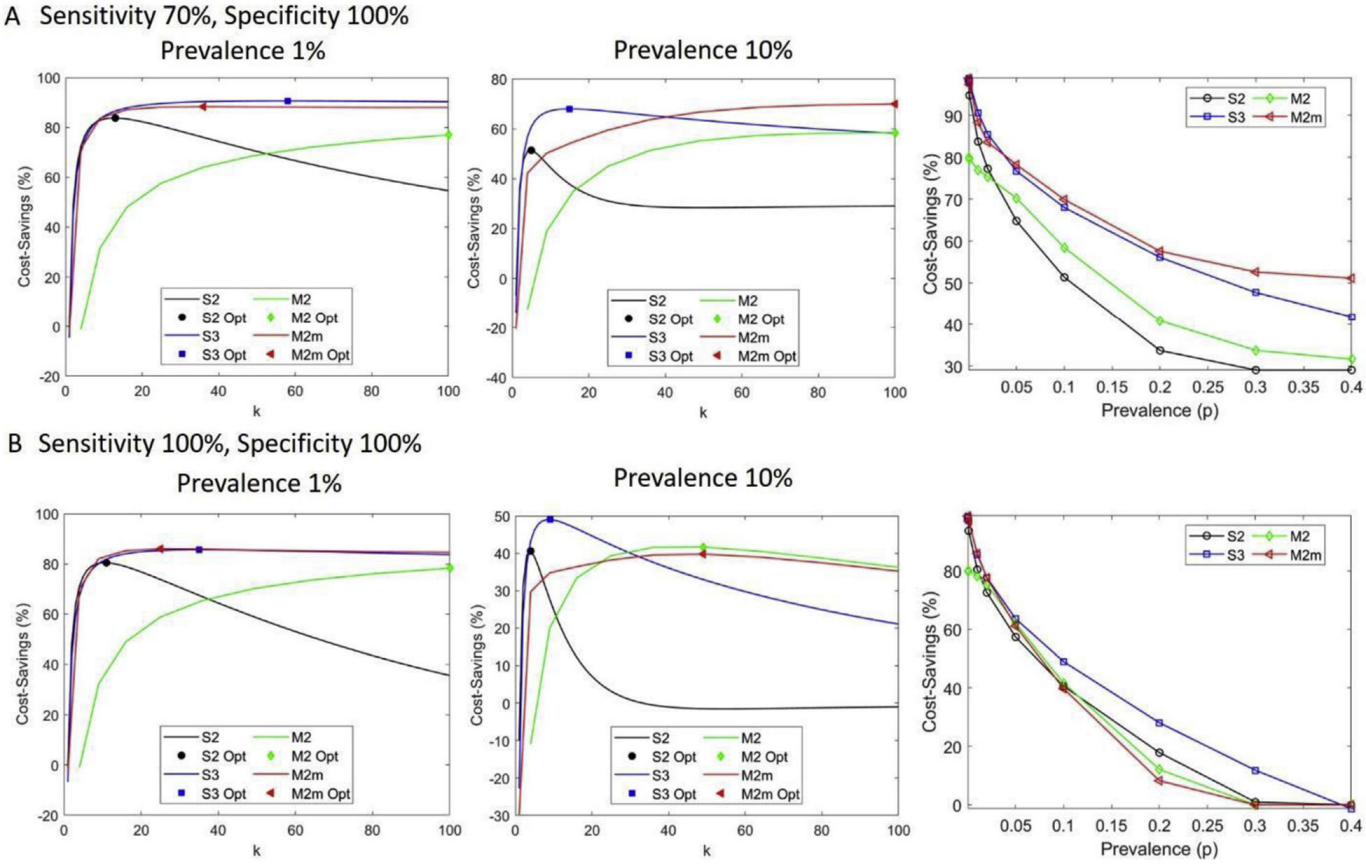
Cost-savings (%) at different test characteristics and prevalence for two-stage hierarchical pool testing (S2), three-stage hierarchical pool testing (S3), matrix-based non-hierarchical pool testing (M2), and matrix-based non-hierarchical pool testing with initial master pool (M2m). (A) Cost-saving with a sensitivity of 70% and specificity of 100%. The first two panels show cost-saving attainable as a function of pool size, k, for two different prevalence rates, 1% and 10%. The symbols indicate the optimal k and cost-saving value. The third panel shows the optimal cost-saving with respect to prevalence. (B) Cost-saving with perfect test characteristics, where the first two panels illustrate cost-saving with respect to pool size for two different prevalences, 1% and 10%, and the third panel shows the optimal cost-saving as a function of prevalence. Here the total number of samples is 100.

**Fig. 4.**
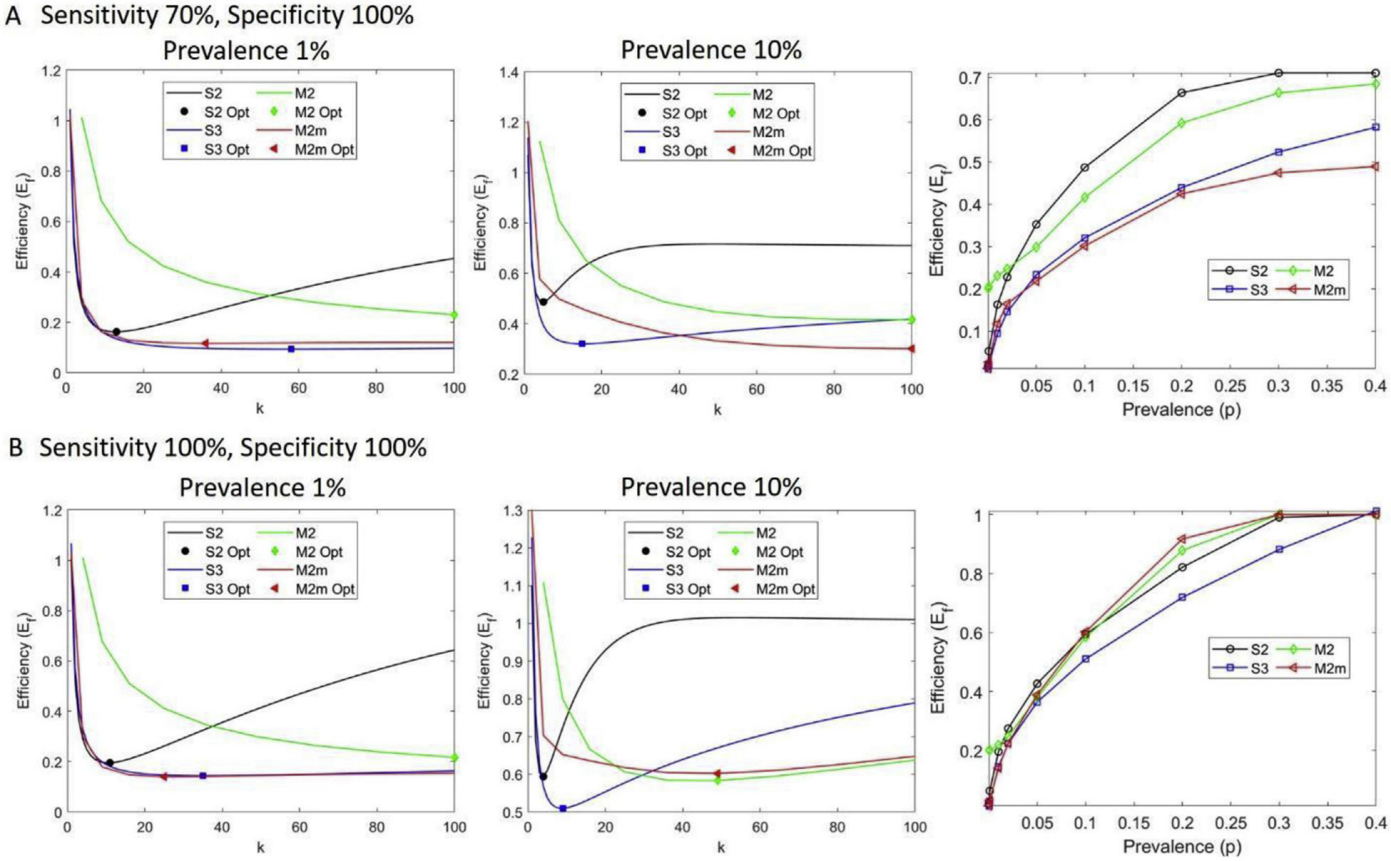
Efficiency (E_f_) at different test characteristics and prevalence for two-stage hierarchical pool testing (S2), three-stage hierarchical pool testing (S3), matrix-based non-hierarchical pool testing (M2), and matrix-based non-hierarchical pool testing with initial master pool (M2m). (A) Pool efficiency for the sensitivity of 70% and specificity of 100%. The first two panels show efficiency as a function of pool size, k, for two different prevalence rates, 1% and 10%. The symbols indicate the optimal k and optimal efficiency attained. The third panel shows the optimal efficiency with respect to prevalence. (B) Efficiency with perfect test characteristics, where the first two panels illustrate efficiency with respect to pool size for two different prevalence rates, 1% and 10%, and the third panel shows the optimal efficiency as a function of prevalence. The smaller the efficiency value the more efficient is the pooling strategy. Here the total number of samples is 100.

Generally, for a low positivity rate, multi-stage greater than two-stage and non-hierarchical matrix-based pool-testing strategies outperform S2 in terms of pool sizes, with pool sizes that are as large as the maximum allowable pool size or samples. In addition, S3 and M2 or M2m have comparable efficiencies (Supplementary Material Table S1). For example, for a sample size of 100 and a maximum allowable pool size of 100, a prevalence of 1%, and a sensitivity of 70% and specificity of 100%, an S2 test has an optimal pool size of 13, efficiency of 16.3% (cost-saving of 83.7%, i.e., about 84% fewer tests will be used), S3 testing has an optimal pool size of 58, efficiency of 9.4% (90.6% cost-saving), and M2m has an optimal pool size of 36 (6 × 6 matrix, in which six samples are in one pool and each sample is tested twice), efficiency of 11.7% (88.3% cost-saving) (Figs. 3 and 4).

Both M2 and S3 strategies are more efficient than S2, although this gap in efficiency narrows considerably at higher prevalence. Considering a similar scenario as before, but with a prevalence of 10%, optimal pool sizes of 5, 15, and 100 (10 × 10 matrix) for S2, S3, and M2m strategies are observed with comparable efficiency of 48.7%, 32.0%, and 30.1%, respectively (Fig. 4). For low prevalence, increasing the complexity of pooling strategies from S2 to M2m is beneficial if only efficiency is important. Both M2 and M2m strategies allow the performance of a larger number of tests compared with the hierarchical strategies S2 and S3, for they can easily be automated as in the case of P-BEST (Pooling Based Efficient SARS-CoV-2 Testing) [19].

**Fig. 5.**
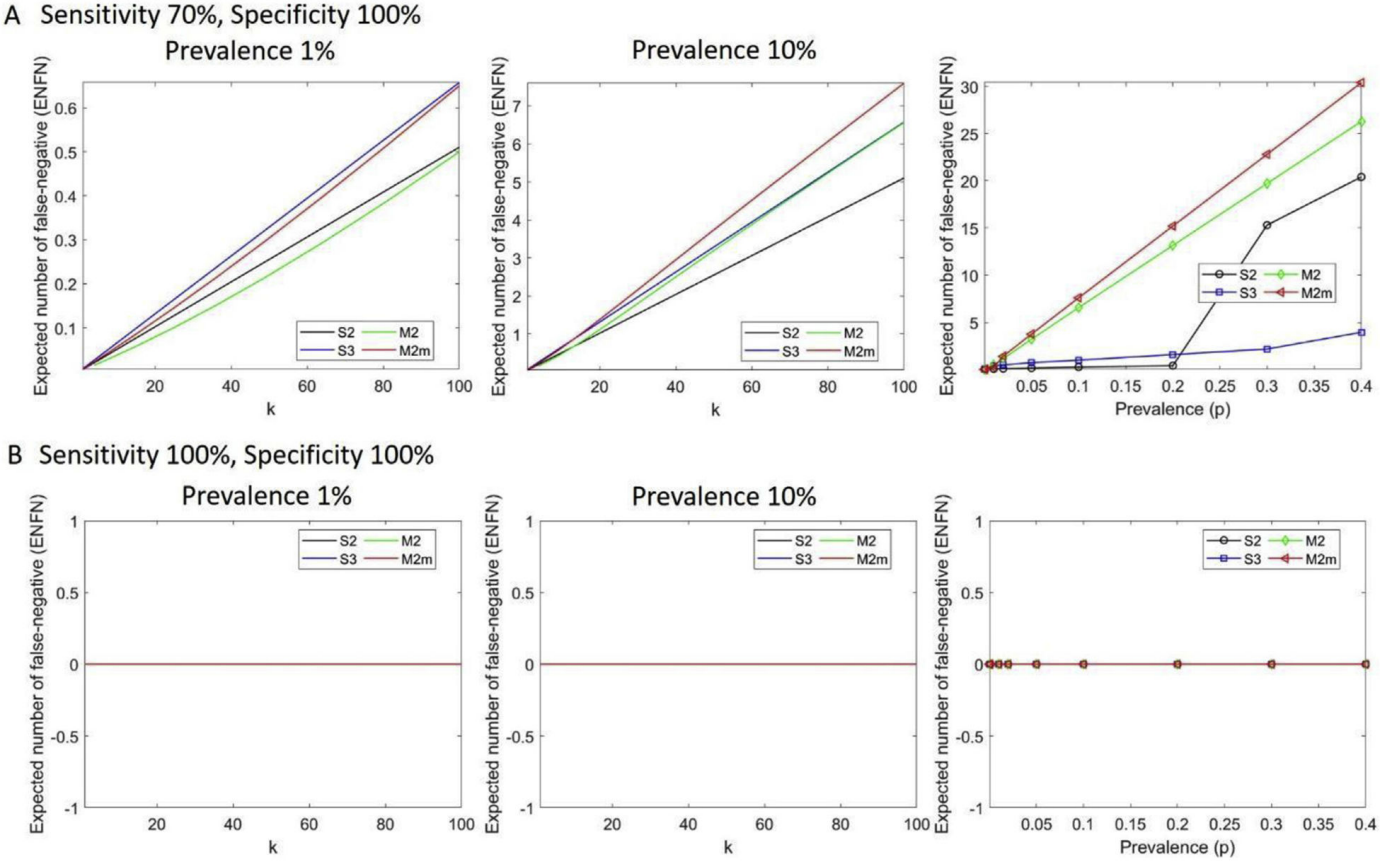
Expected number of false negatives (ENFNs) at different test characteristics and prevalence for two-stage hierarchical pool testing (S2), three-stage hierarchical pool testing (S3), matrix-based non-hierarchical pool testing (M2), and matrix-based non-hierarchical pool testing with initial master pool (M2m). (A) Expected number of false negatives as a function of pool size, k, at two different prevalence rates,1% and 10% (first two panels), and as a function of prevalence (third panel) under realistic test characteristics with 70% sensitivity and 100% specificity. (B) Expected number of false negatives under perfect test characteristics at two different prevalence rates, 1% and 10% (first two panels), and as a function of prevalence (third panel). With perfect sensitivity and specificity, the number of false negatives is zero. Here the total number of samples is 100.

However, the larger the pool size, the higher the probability of false negatives. For instance, at a prevalence of 20% (sensitivity of 70% and specificity of 100%), S2 has an optimal pool size of 4 with a cost-saving of 34% and 0.41 false-negative results (less than one false-negative case per 100 samples), while M2m has an optimal pool size of 100 with 58% cost-saving. However, M2m has 15 expected false negatives. As a result, when prevalence increases, matrix-based pool testing is no longer efficient, and false negatives increase despite a higher number of the optimal pool size (Fig. 5). With perfect sensitivity and specificity, the number of false negatives is zero (Fig. 5B). For a test with a 70% sensitivity and 100% specificity, a false-negative number >1 is observed at a prevalence of 30% for S2, at 20% for S3, and at 2% for M2m. The optimal pool size is a decreasing function of prevalence until it is no longer effective compared with single individual testing (Supplementary Material Table S1).

Although there is a differential performance based on efficiency, a multi-stage of more than three can be practically difficult to implement. The S2 strategy is the simplest to practically execute and has the benefit of reducing turnaround time for reporting positive results compared to S3 and matrix-based approaches, as they may involve additional logistical constraints, ranging from software support needed to guide laboratory operation to the number of potential samples needed to perform a large number of repeat tests. However, reductions in assay specificity may reduce the effectiveness of S2 algorithms, while S3 and matrix-based approaches are more robust and less sensitive to changes in assay specificity.

There are several modifications to these classical pool-testing strategies to further improve efficiency and minimize dilution effect and misclassification of the results. Escobar et al. [20] employed machine learning, using clinical or demographic data, to rearrange samples into pools based on their probability of testing positive for COVID-19. Samples with high probabilities are tested individually, and low-probability samples undergo the S2 strategy. The authors claim that their approach enhances efficiency and also extends the validity region. However, it is not clear how the approach improved efficiency, as the performance of the smart pooling and classical S2 is comparable for prevalence >20%, and the efficiencies for the smart pooling are slightly higher than those of S2. Also, the authors do not show the performance of other operating characteristics, such as false-negative rates and dilution effects.

Shental et al. [19] employed a combinatorial (non-adaptive/non-hierarchical) pooling strategy based on the compressed sensing method, called P-BEST, to improve efficiency. However, the eight-fold improvement in testing efficiency was shown only for a prevalence <1.3%. At such prevalence, it is not obvious whether P-BEST outperforms a simple matrix-based pool testing. In addition, due to the need to split each individual sample into multiple pools, and a computer algorithm that itself may fail to correctly identify all positive individuals, this method may lead to higher false rates (both positive and negative).

## Practical execution of pool testing

### Practical execution in general

Diagnostic tests identify pathogen- and disease-specific molecules extracted from clinical samples. Practically, mixed samples collected from different individuals can be pooled before or after the extraction step. Although the practical execution described herein is focused mainly on COVID-19, the majority of the concepts can be quickly adapted to general circumstances and other infectious diseases if the test sensitivity is high while the prevalence is low. We investigated pooling of swabs, saliva, and gargle samples in COVID-19 practice. Other types of samples can be explored in research if they are shown to be promising for diagnostic use.

### Practical execution in COVID-19

Currently the reference standard for clinical diagnostic detection of severe acute respiratory syndrome coronavirus 2 (SARS-CoV-2) remains real-time reverse transcription-polymerase chain reaction (RT-PCR), although antigen or antibody tests have been authorized for rapid point-of-care COVID-19 testing. A recent study explored the use of anti-SARS-CoV-2 antibody-based pool testing as a complementary surveillance tool and for the identification of possible convalescent plasma donors [21]. However, antigen or antibody serology pool testing is not recommended for diagnostic or screening testing of COVID-19 (e.g. in workplaces or schools). First, research shows that the average sensitivity is low to moderate, at 56.2% for antigen test [22] and 84.5% for IgM or 91.6% for IgG antibody tests [23] for individuals who were confirmed positive by SARS-CoV-2 nucleic acid diagnostics. Second, SARS-CoV-2 antibodies, especially IgG, remain positive for several months [24], which presumably leads to high prevalence, rendering pool testing less effective. Nevertheless, COVID-19 serology pool testing may be feasible for assessing community- or population-level infection rates and trends after interventions have been implemented. Such efforts could, for example, utilize residual serum specimens or samples collected for other purposes and handled by large laboratories.

**Fig. 6.**
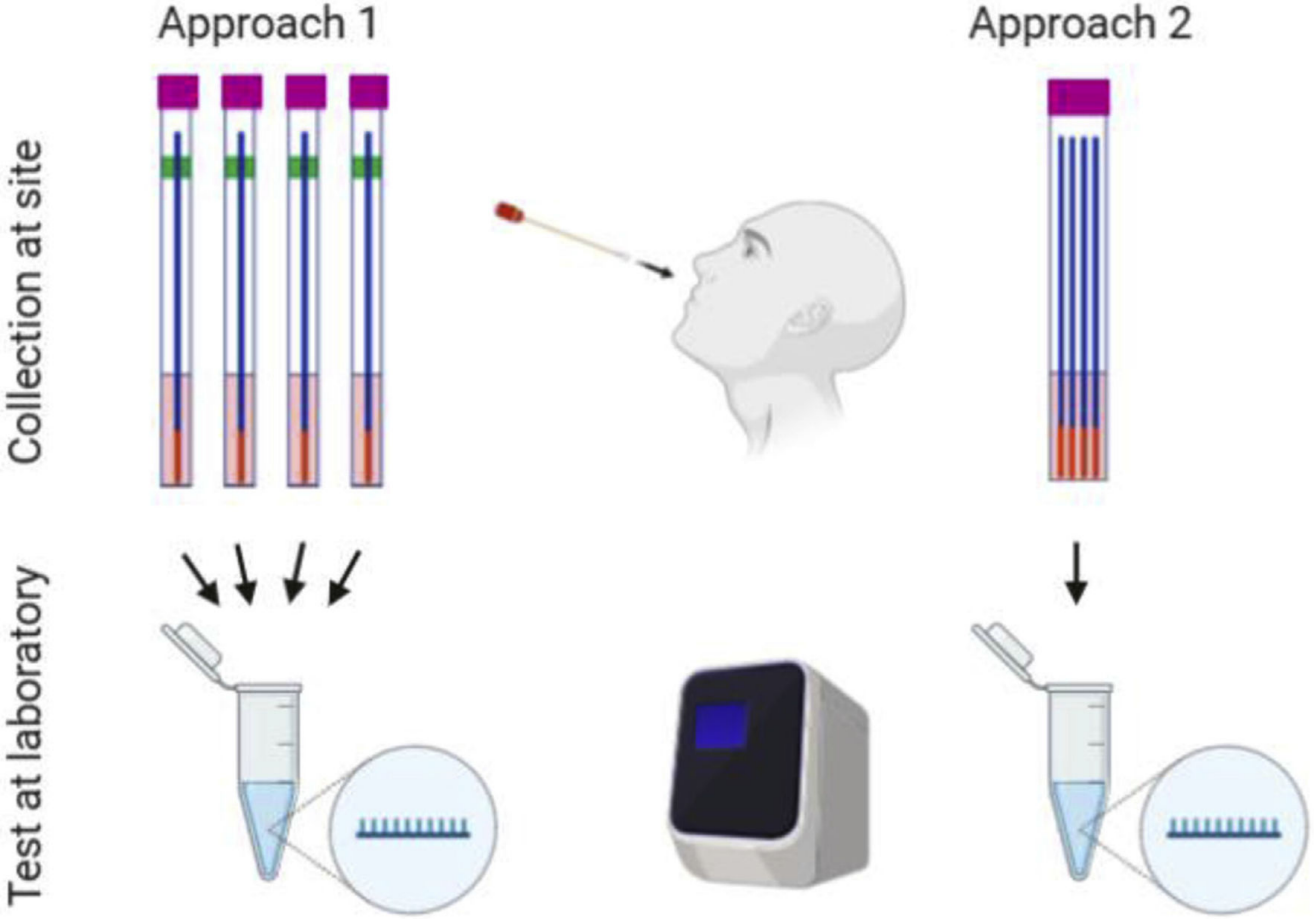
Practical execution of pool testing. In Approach 1 (‘media pooling’), individual swab samples are collected on site, transported to the testing laboratory, and media are mixed in the laboratory into pools, which are then tested. In Approach 2 (‘swab pooling’), swab samples are pooled together into the same container at the collection site and transported to the testing laboratory, where the media of the pooled swabs are tested.

There are two approaches to performing RT-PCR-based SARS-CoV-2 pool testing. In approach 1, individual samples are collected on site in viral transportation medium (VTM). The VTM is mixed in the lab into pools, which are then tested by RT-PCR (Fig. 6 Approach 1). Pooling of samples before RNA extraction has been shown to be either not significantly different [25] or preferable [26] as compared to pooling samples at the RNA level.

Another option is to pool all samples together into VTM in the same collection container (Fig. 6 Approach 2). Schmidt et al. evaluated a combination of the two approaches by incubating swabs in a single-sample tube and then again in a multiple-swab tube [27]. Their results showed that the analytical sensitivity was constant up to a total number of 50 swabs. This method does not lead to significantly different cycle threshold values between single swab and multiple swabs.

## Limitations

### General consideration

Pool testing poses several challenges independently of specific clinical applications. An obvious question is where pooling should happen. There are two main options: at the site of collection or in the laboratory. To the best of our knowledge, pooling at the collection site is not practised at most places. The major challenge for pooling is the dilution effect if the test is sensitive to sample dilution. As a result, tests will likely have higher false-negative rates compared with individual testing. In addition, considerations before implementing pool testing should be given to adequate sample collection, quality of collection material, efficient and validated sample preparation protocols, and a highly sensitive detection method.

Apart from practical and execution considerations, economic aspects of pool testing deserve mentioning. In most instances, testing is reimbursed on a per-test basis. Since pool testing will most likely require fewer tests, the costs to payors will be reduced. However, at the same time, pool testing will impact laboratories' revenues. This creates a disincentive that may impede the implementation of pool-testing strategies in laboratories. Transparent negotiations between payors, laboratories, and healthcare providers are necessary to address this issue.

### Specific consideration related to SARS-CoV-2

Asymptomatic SARS-CoV-2-positive patients on average have lower viral copy numbers than symptomatic patients [28]. For symptomatic patients, viral load is highest around the onset of symptoms and decreases shortly after. When a low concentration of SARS-CoV-2 virus is diluted into a pool of negative samples there will be a risk that levels are close to the limit of detection, thus increasing false-negative rates. Therefore, an obvious issue for SARS-CoV-2 pool testing using Approach 1 (Fig. 6) is the reduced sensitivity due to sample dilution. In principle, pooling one positive sample with seven negative samples will cause eight-fold viral dilution in the pool, which equates with three increased cycles of RT-PCR. A pool of 32 samples will increase the cycle number by 5. False negatives are inevitable if samples' viral loads are close to the detection limit. In fact, false-negative results are always a concern, even for individual testing. Research has shown that the probability of a false-negative result in an infected person decreases from 100% on day 1 to 67% on day 4 after exposure [29]. On the day of symptom onset, typically 4 days after exposure, the median false-negative rate is 38%, which further decreases to 20% on day 8 (3 days after symptom onset). This time point may be the optimal time for RT-PCR testing since the median false-negative rate increases from day 9 onward to 66% on day 21 [27]. The fact that pool testing will likely have even higher false-negative rates is a concern, especially if the goal is to screen individuals at an early stage of infection [29].

Carrier nucleic acids are commonly used to increase the yield of extraction, especially for biological specimens with a small amount of target RNA/DNA. Interestingly, Lohse and colleagues found lower cycle threshold values in pooled samples compared with individual samples and hypothesized that this was due to the carrier effect of potentially higher cellular RNA content [30]. However, other studies have not been able to confirm this effect [31,32]. Different swab systems may cause the discrepancies between studies [33].

Nasopharyngeal (NP) and oropharyngeal (OP) swab specimens are widely used across the world for SARS-CoV-2 RNA testing. They have been recognized as the most sensitive methods if collection is done properly. But both samplings must be done by healthcare professionals, limiting their application to designated venues only. NP swab samples are uncomfortable and put healthcare workers at increased risk of contagion. Self-tests would reduce exposure risks for others; however, at least one healthcare professional should be present to observe a proper swab specimen collection. Therefore, new advances in sampling techniques and collection of alternative specimens address these challenges and are currently being explored. For example, it has been shown that testing in saliva may be as sensitive as NP swabs, with less variation in levels of SARS-CoV-2 RNA in the former [34]. Pooling saliva provides a simple method to expand SARS-CoV-2 testing capacity [35,36] although it is not as sensitive as individual swab testing [37]. Besides saliva sampling, saline mouth rinse/gargle sampling has been shown to be another reliable testing source for SARS-CoV-2 [38]. These and other sample types will be continuously explored for pool testing in research settings and, if they are shown to be promising, further validated for clinical diagnostics.

Pooling is logistically more challenging than individual testing to implement at the collection site, and there is always the potential for accidental swapping of samples after collection. Another drawback of this method is that if a pool tests positive, individual samples from this pool need to be recollected to identify the positive cases. A possible way to avoid recollection is to use a special swab that can be split into two parts: one half goes to a pool and the other half remains in an individual collection container.

## Other clinical applications of pool testing

Existing and emerging communicable diseases have the potential to threaten the fabric of society. In order to prevent future outbreaks from spreading, pool testing is an efficient method for tracing cases rapidly. In particular, outbreaks of methicillin-resistant *Staphylococcus aureus* (MRSA) pose substantial problems to hospitals. Pool testing has been used in such circumstances, and it has been shown that pooling different kinds of swabs together does not decrease sensitivity. The performance of the Xpert MRSA PCR assay on pooled nose, groin, and throat swabs was compared to culture. In one study, 5555 pooled samples were collected from 3774 patients; the sensitivity (0.78) and specificity (0.99) were similar to the results from published studies on separated nose or other specimens [39]. Another study reported similar results between samples from non-nasal sites and samples from multiplesite pooled swabs, showing a potential for reducing laboratory work and cost [40]. Of note, screening swabs should also be taken from multiple sites, as testing with only nasal swabs failed to identify 24% of MRSA-colonized patients [41].

## Outlook and conclusion

The speed of testing and reporting is key to containing infectious diseases. To inform reopening strategies, the number of tests needs to be increased drastically. Pool testing will relieve overwhelmed laboratories and improve speed of test reporting. Pool testing is a much more efficient COVID-19 testing process with the potential to bring the pandemic under control worldwide, and thus has the potential to save lives. Financial considerations need to be addressed to make pool testing an economically viable option.

Adoption of a pool-testing strategy as an effective screening tool for COVID-19 needs to be carefully evaluated. For example, binary splitting applies a binary method to hierarchical multi-stage strategies, such as S2 and S3, where a pool is split into two sets of size k/2, which are then sampled and tested. Positive results are recursively subdivided until each individual case has been found. This procedure can be optimized by using binary search algorithms at each subdivision, and by choosing optimal initial pool sizes based on prevalence as is done in S2, S3, M2 and M2m [42,43]. Similarly, a binomial distribution-based decision-tree algorithm can also be used to obtain optimal pool size according to optimization of the expected number of remaining tests [42,44]. Other high-dimensional pool-testing algorithms have also been proposed. Mutesa and colleagues proposed a proof-of-concept based on hypercube pool-testing strategies where four-way pooling is considered in order to increase testing of low-risk populations [45]. The authors showed that pooling samples for SARS-CoV-2 molecular detection can efficiently be performed without sacrificing substantial accuracy or specificity at lower prevalence (i.e. for prevalence <8%). Like S2, S3, M2, M2m, and other modified pooling strategies, a pooling approach proposed by Mutesa and colleagues also improves efficiency of the pooled samples. However, as shown in the Supplementary Material (Table S1), as the complexity of the pooling strategy increases, we also observe increases in both false-negative and false-positive rates.

Further improvements can be made to increase pool size and efficiency without sacrificing the various operating characteristics, such as probability and the expected number of false positives and false negatives and positive and negative predictive values. A robust pooling strategy can be employed in one of the pool testing methods (S2, S3, M2, and M2m) by considering the uncertainties associated with prevalence and test errors. Similarly, a dynamic pool testing with an adaptive design moving between pooling strategies or selecting optimal pool size could be used. Similar to smart pooling [20], one can incorporate (dynamic) pre-test likelihood to increase efficiency and reduce false-negative rates. Other alternatives—such as the prevalence spiralling method in conjunction with non-hierarchical matrix-based pooling strategies—can also be used, where high-risk individuals are clustered on one side of the array. Recently, Daon et al. proposed a pooling strategy that uses a Bayesian D-Optimal pooling experimental design by maximizing the mutual information between the data and the infection states [46]. The authors report lower rates and fewer test utilizations.

Our answer to the question posed in the title of this review is leaning towards ‘solution’. We acknowledge that there are problems which need to be addressed, especially regarding the logistics. Focusing on these will help to advance pool testing and mitigate the onslaught of the current and future pandemics.

## Supplementary Material

supplement

## Figures and Tables

**Table 1:** Applied cases of pool-testing strategies for coronavirus disease 2019 (COVID-19) in different countries with regard to the type of test, pool size, total number of tests, and the location used. Although detection of positive samples is attainable for a pool size of up to 48 samples, most used pool sizes are less than ten samples. Applied locations are mainly low-acuity settings (hospital, island, care home etc.), including the case used in Wuhan, China, when the country progressed into a post-lockdown phase. The successful application of pool testing in various settings suggests an economical potential for regular screening to enable safer in-person interactions

Country	Type of test	Pool size (samples)	Total number of tests	Applied location	Reference
Australia	NP	2, 4, 8	Over 29 000^a^	Lower acuity settings	[47]
Austria	—	—	—	University, Hospital	Personal communication Franz Allerberger
Brazil	NP	4, 8, 16, 32	6096	Industrial Federation	[48]
Brazil	NP	16	18 922	Hospital	[49]
China	OP	5	2.3 million	The City of Wuhan	[50]
Ecuador	NP	3	Over 2000/month^b^	Laboratory Galapagos Islands	[51]
Germany	NP	4-30	1191^c^	Medical Center	[30]
Germany	NP	10	700^d^	Laboratory	[52]
India	NP/OP	5	545	3 Central Indian Districts	[53]
India	NP/OP	5 or 10	19 560^e^	Communities with varying prevalence of population	[54]
Israel	Nasal + OP/NP^f^	8	26 576	Hospital, community, essential industries	[55]
Israel	NP/OP	Up to 48	30 000/week	Insurance, diagnostic company	[19], personal communication Tomer Hertz
Malaysia	NP/OP	5,10	2732	Close contacts of confirmed COVID-19 cases, collected in community and primary care clinics	[56]
Pakistan	NP	6	83^g^	Dental clinics	[57]
Rwanda	OP	20	—	Nationwide and required for all air passengers	[45]
Spain	NP	20^h^	25 386	Care homes	[58]
Spain	NP	5	4475	Hospital	[59]
USA	NP	5	60	Laboratory	[8]
USA	NP	up to 10	Over 10 000	Army and other service units	[60]

NP, nasopharyngeal swab; OP, oropharyngeal swab.

a A total of 10 312 and 19 388 clinical samples were pooled for testing in March and May 2020, respectively.

b One hundred and fourteen clinical specimens were selected for the evaluation study. The protocol was successfully used to test over 2000 subjects within a period of a month during COVID-19 surveillance at Galapagos Islands.

c From 13th to 21st March 2020, pool-testing of 1191 samples required only 267 tests and detected 23 positive individuals (prevalence 1.93%).

d Seven hundred samples were tested within the period 7th February 2020 to 10th October 2020.

e Between April and June 2020, 4620 samples were tested in 462 pools of ten and 14 940 samples in 2990 pools of five.

f A single swab was collected for combined deep nasal and oropharyngeal collection from the same patient.

g From 28th May 2020 to 20th July 2020, 194 dental patients were advised for SARS-CoV-2 testing; 111 patients chose not to get tested, and the remaining 83 were pool tested.

h The prevalence and distribution of positive detection in care homes were calculated to determine a pool size of 20 samples and a sub-pool of five samples (used when a pool of 20 tested positive).
