# Supplementary material for: Sample pooling: burden or solution?
Source: Clin Microbiol Infect. Author manuscript; Available in PMC 2022 Sep 15. (PMC9477502; doi:10.1016/j.cmi.2021.04.007)
Supplement: supplement [file NIHMS1834423-supplement-supplement.docx]

**Table S1: Optimal Pool Size (k), Efficiency (Ef), and Expected Number of False Negatives (ENFN) with respect to prevalence and test errors for a maximum allowable pool of 100 individuals, where specificity is 100%.** Ef is defined as the expected number of tests required per individual specimen, where Ef of individual testing is 1, while Ef of less than 1 indicates that the pooling strategy will require fewer tests on average than individual testing.

| **Sensitivity** |  | **S2** | | |  | **S3** | | |  | **M2** | | | |  | | **M2m** | | | |
| --- | --- | --- | --- | --- | --- | --- | --- | --- | --- | --- | --- | --- | --- | --- | --- | --- | --- | --- | --- |
|  | **p** | **k** | **Ef** | **ENFN** |  | **k** | **Ef** | **ENFN** |  | **k** | **Ef** | **ENFN** |  | | **k** | | **Ef** | **ENFN** |  |
| 1 | 0.0001 | 100 | 0.020 | 0 |  | 100 | 0.012 | 0 |  | 100 | 0.200 | 0 |  | | 100 | | 0.012 | 0 |  |
|  | 0.001 | 32 | 0.063 | 0 |  | 100 | 0.028 | 0 |  | 100 | 0.201 | 0 |  | | 100 | | 0.030 | 0 |  |
|  | 0.01 | 11 | 0.196 | 0 |  | 35 | 0.144 | 0 |  | 100 | 0.217 | 0 |  | | 25 | | 0.140 | 0 |  |
|  | 0.02 | 8 | 0.274 | 0 |  | 23 | 0.223 | 0 |  | 100 | 0.247 | 0 |  | | 16 | | 0.224 | 0 |  |
|  | 0.05 | 5 | 0.426 | 0 |  | 13 | 0.364 | 0 |  | 81 | 0.380 | 0 |  | | 100 | | 0.389 | 0 |  |
|  | 0.1 | 4 | 0.594 | 0 |  | 9 | 0.510 | 0 |  | 49 | 0.583 | 0 |  | | 49 | | 0.602 | 0 |  |
|  | 0.2 | 3 | 0.821 | 0 |  | 6 | 0.719 | 0 |  | 25 | 0.879 | 0 |  | | 25 | | 0.917 | 0 |  |
|  | 0.3 | 3 | 0.990 | 0 |  | 6 | 0.881 | 0 |  | 1 | 1 | 0 |  | | 1 | | 1 | 0 |  |
|  | 0.4 | 1 | 1 | 0 |  | 1 | 1 | 0 |  | 1 | 1 | 0 |  | | 1 | | 1 | 0 |  |
|  | | | | | | | | | | | | | | | | | | | |
| 0.95 | 0.0001 | 100 | 0.019 | 0.001 |  | 100 | 0.012 | 0.001 |  | 100 | 0.200 | 0.001 |  | | 100 | | 0.012 | 0.001 |  |
|  | 0.001 | 33 | 0.061 | 0.003 |  | 100 | 0.026 | 0.014 |  | 100 | 0.202 | 0.006 |  | | 100 | | 0.030 | 0.011 |  |
|  | 0.01 | 11 | 0.190 | 0.011 |  | 38 | 0.135 | 0.054 |  | 100 | 0.220 | 0.104 |  | | 25 | | 0.138 | 0.029 |  |
|  | 0.02 | 8 | 0.267 | 0.016 |  | 25 | 0.210 | 0.071 |  | 100 | 0.246 | 0.253 |  | | 100 | | 0.218 | 0.340 |  |
|  | 0.05 | 5 | 0.415 | 0.024 |  | 14 | 0.341 | 0.100 |  | 100 | 0.363 | 0.707 |  | | 100 | | 0.354 | 0.922 |  |
|  | 0.1 | 4 | 0.577 | 0.039 |  | 9 | 0.477 | 0.128 |  | 49 | 0.555 | 0.692 |  | | 49 | | 0.546 | 0.902 |  |
|  | 0.2 | 3 | 0.797 | 0.059 |  | 7 | 0.670 | 0.200 |  | 25 | 0.834 | 0.705 |  | | 36 | | 0.826 | 1.334 |  |
|  | 0.3 | 3 | 0.957 | 0.088 |  | 6 | 0.818 | 0.257 |  | 1 | 1 | 1.068 |  | | 1 | | 1 | 5.565 |  |
|  | 0.4 | 100 | 0.960 | 3.900 |  | 6 | 0.937 | 0.342 |  | 1 | 1 | 5.705 |  | | 1 | | 1 | 7.420 |  |
|  | | | | | | | | | | | | | | | | | | | |
| 0.8 | 0.0001 | 100 | 0.018 | 0.004 |  | 100 | 0.011 | 0.005 |  | 100 | 0.200 | 0.002 |  | | 100 | | 0.012 | 0.004 |  |
|  | 0.001 | 36 | 0.056 | 0.013 |  | 100 | 0.023 | 0.049 |  | 100 | 0.204 | 0.025 |  | | 100 | | 0.028 | 0.040 |  |
|  | 0.01 | 12 | 0.174 | 0.043 |  | 48 | 0.110 | 0.234 |  | 100 | 0.227 | 0.363 |  | | 36 | | 0.127 | 0.155 |  |
|  | 0.02 | 9 | 0.244 | 0.065 |  | 31 | 0.170 | 0.303 |  | 100 | 0.245 | 0.853 |  | | 100 | | 0.185 | 1.082 |  |
|  | 0.05 | 6 | 0.379 | 0.108 |  | 18 | 0.275 | 0.439 |  | 100 | 0.320 | 2.401 |  | | 100 | | 0.265 | 2.921 |  |
|  | 0.1 | 4 | 0.525 | 0.144 |  | 12 | 0.381 | 0.586 |  | 64 | 0.474 | 3.100 |  | | 81 | | 0.392 | 4.775 |  |
|  | 0.2 | 4 | 0.722 | 0.288 |  | 9 | 0.529 | 0.878 |  | 49 | 0.693 | 4.776 |  | | 64 | | 0.574 | 7.556 |  |
|  | 0.3 | 100 | 0.810 | 10.800 |  | 9 | 0.637 | 1.318 |  | 100 | 0.805 | 14.640 |  | | 100 | | 0.654 | 17.712 |  |
|  | 0.4 | 100 | 0.810 | 14.400 |  | 10 | 0.719 | 1.952 |  | 100 | 0.832 | 19.520 |  | | 100 | | 0.676 | 23.616 |  |
|  | | | | | | | | | | | | | | | | | | | |
| 0.7 | 0.0001 | 100 | 0.017 | 0.005 |  | 100 | 0.011 | 0.007 |  | 100 | 0.200 | 0.004 |  | | 100 | | 0.012 | 0.006 |  |
|  | 0.001 | 39 | 0.052 | 0.020 |  | 100 | 0.020 | 0.066 |  | 100 | 0.204 | 0.038 |  | | 100 | | 0.026 | 0.057 |  |
|  | 0.01 | 13 | 0.163 | 0.066 |  | 58 | 0.094 | 0.381 |  | 100 | 0.231 | 0.499 |  | | 36 | | 0.117 | 0.213 |  |
|  | 0.02 | 9 | 0.227 | 0.092 |  | 37 | 0.145 | 0.486 |  | 100 | 0.247 | 1.143 |  | | 100 | | 0.164 | 1.400 |  |
|  | 0.05 | 6 | 0.352 | 0.153 |  | 22 | 0.233 | 0.723 |  | 100 | 0.298 | 3.209 |  | | 100 | | 0.218 | 3.747 |  |
|  | 0.1 | 5 | 0.487 | 0.255 |  | 15 | 0.320 | 0.986 |  | 100 | 0.416 | 6.558 |  | | 100 | | 0.301 | 7.590 |  |
|  | 0.2 | 4 | 0.663 | 0.408 |  | 12 | 0.439 | 1.577 |  | 100 | 0.592 | 13.139 |  | | 100 | | 0.424 | 15.197 |  |
|  | 0.3 | 100 | 0.710 | 15.300 |  | 11 | 0.523 | 2.168 |  | 100 | 0.663 | 19.710 |  | | 100 | | 0.474 | 22.797 |  |
|  | 0.4 | 100 | 0.710 | 20.400 |  | 15 | 0.582 | 3.942 |  | 100 | 0.684 | 26.280 |  | | 100 | | 0.489 | 30.396 |  |

S2 - 2-stage hierarchical pool testing (Dorfman's), S3 - 3-stage hierarchical pool testing, M2 - matrix-based non-hierarchical pool testing, M2m - matrix-based hierarchical pool testing with a master pool. M2m is similar to M2, where a master pool is tested first before implementing M2. If a master pool is positive, then the M2 strategy is performed to test row and column pools as in M2.
